# 5azadC treatment upregulates miR-375 level and represses HPV16 E6 expression

**DOI:** 10.18632/oncotarget.17575

**Published:** 2017-05-02

**Authors:** Adrien Morel, Aurélie Baguet, Jérôme Perrard, Caroline Demeret, Elise Jacquin, David Guenat, Christiane Mougin, Jean-Luc Prétet

**Affiliations:** ^1^ EA3181, Université Bourgogne Franche-Comté, LabEx LipSTIC ANR-11-LABX-0021, Besançon, France; ^2^ Département de Virologie, Institut Pasteur, Unité de Génétique Moléculaire des Virus à ARN, CNRS UMR 3569, Université Paris Diderot, Sorbonne Paris Cité, Paris, France; ^3^ Signalling Department, The Babraham Institute, Babraham Research Campus, Cambridge, United Kingdom; ^4^ Centre Hospitalier Régional Universitaire, Besançon, France; ^5^ Department of Medicine, Division of Oncology, Stanford Cancer Institute, Stanford University, Stanford, California, USA

**Keywords:** HPV, DNA methylation, E6, miRNA, 5azadC

## Abstract

High-risk human papillomaviruses are the etiological agents of cervical cancer and HPV16 is the most oncogenic genotype. Immortalization and transformation of infected cells requires the overexpression of the two viral oncoproteins E6 and E7 following HPV DNA integration into the host cell genome. Integration often leads to the loss of the E2 open reading frame and the corresponding protein can no longer act as a transcriptional repressor on p97 promoter. Recently, it has been proposed that long control region methylation also contributes to the regulation of E6/E7 expression.

To determine which epigenetic mechanism is involved in HPV16 early gene regulation, 5-aza-2′-deoxycytidine was used to demethylate Ca Ski and SiHa cell DNA. Decreased expression of E6 mRNA and protein levels was observed in both cell lines in an E2-independent manner. E6 repression was accompanied by neither a modification of the main cellular transcription factor expression involved in long control region regulation, nor by a modification of the E6 mRNA splicing pattern. In contrast, a pronounced upregulation of miR-375, known to destabilize HPV16 early viral mRNA, was observed. Finally, the use of miR-375 inhibitor definitively proved the involvement of miR-375 in E6 repression. These results highlight that cellular DNA methylation modulates HPV16 early gene expression and support a role for epigenetic events in high-risk HPV associated-carcinogenesis.

## INTRODUCTION

Among the 170 HPV described in 2013 [1], 12 high-risk HPV (hrHPV) are classified as carcinogenic to humans by the IARC [2]. High-risk HPV are the etiologic agents of cervical cancer and are also responsible for the majority of anal cancers and part of head and neck, vaginal, vulvar and penile cancers. HPV16 is the most carcinogenic genotype, found in 61% of cervical cancers according to a retrospective cross-sectional worldwide study [3], in 73.4% of anal cancers [4] and 30.9% of oropharyngeal cancers [5].

The HPV16 genome is a circular double-stranded DNA. It harbors 8 open reading frames (ORFs) encoding early (E1, E2, E4, E5, E6 and E7) and late (L1 and L2) viral proteins and a long control region (LCR) containing transcription control sequences. The LCR presents numerous binding sites for cellular transcription factors known to activate (TATA binding protein, Sp1, AP-1 and NFI) or repress (YY1) transcription of viral early genes [6]. It also exhibits four E2 Binding Sites (E2BS) with common consensus sequence 5′-ACCG(N)_4_CGGT-3′ and different affinities for E2 [7]. The two E2BS closest to the TATA box (proximal E2BS) are the main sites involved in the repression of p97 promoter activity by E2. The repressor activity of E2 is linked to its ability to displace cellular transcriptional activators from their binding site due to steric hindrance [8].

The p97-mediated transcription produces polycistronic mRNAs undergoing alternative splicing [9]. In the absence of splice-site recognition in the E6 ORF, the full length E6 (E6Fl) protein, showing oncogenic properties, is produced. In contrast, the recognition of alternative splice sites generates transcripts potentially encoding truncated E6 proteins such as E6*I and E6*II [10], two isoforms whose function remains poorly understood [11, 12].

HPV16 DNA integration into the host cell genome is thought to play a pivotal role in HPV-associated carcinogenesis. Integration generally results in the disruption of the E2 ORF, thereby preventing the repressive function of E2 on p97 promoter. This leads to an upregulation of E6 and E7, two viral oncoproteins that deregulate the p53 and p105Rb pathways respectively [13, 14]. Consequently, E6 and E7 induce the immortalization and transformation of infected cells.

Some authors have proposed that sequence variations or changes of the LCR methylation status could contribute to the regulation of E6/E7 expression [15]. In this regard, we and others previously showed CpG dinucleotide methylation of HPV16 E2BS in precancerous and cancerous lesions of the cervix [16–20]. However, upregulation of E6 and E7 is not only due to the transcriptional activation of p97, but also to the increased stability of early viral mRNA transcribed from integrated HPV16 genomes [21].

Recently, several studies reported an aberrant expression profile of 341 miRNAs [22] in HPV-associated carcinomas and the detection of miR-424/miR-375/miR-218 from cervical smears has been proposed for cervical cancer screening [23]. Furthermore, miR-375, a tumor suppressor miRNA known to target HPV16 E6/E7 mRNA [24] is downregulated in cervical carcinomas compared to normal tissues or precancerous lesions [25, 26] thanks to promoter methylation [27].

In this study, the demethylating agent 5-aza-2′deoxycytidine (5azadC) was used to treat the HPV16-positive cervical carcinoma Ca Ski and SiHa cell lines. Our objective was to characterize the impact of DNA demethylation on early viral gene expression.

## RESULTS

### 5azadC treatment decreases HPV16E6 expression in Ca Ski and SiHa cells in E2-independent manner

In order to determine the impact of 5azadC on endogenous HPV16 E6 protein levels, time-course and dose-response experiments were conducted in Ca Ski and SiHa cells. These cell lines differ in their status regarding HPV16 genome integration. The Ca Ski cells harbor up to 500 copies of integrated HPV16 genomes, most copies being hyper-methylated. In contrast, SiHa cells harbor one to two integrated viral genomes per cell [28]. Interestingly, from 24h to 96h, E6 protein levels gradually decreased in both cell lines treated with 5azadC. In parallel, an accumulation of p53 protein was observed (Supplementary Figure 1). According to these results, the treatment condition used to monitor the effect of 5azadC on E6 expression was set at 0.25 μM for 96h.

**Figure 1:**
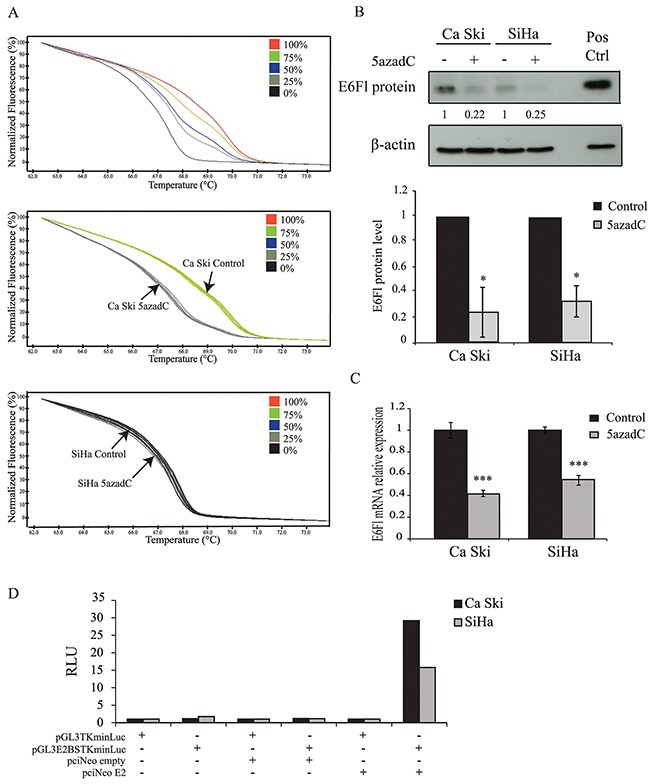
Effect of 5azadC treatment on proximal E2BS methylation and E6 expression **(A)** Normalized HRM curves obtained from standards containing 0%, 25%, 50%, 75% and 100% methylated target (top) and from Ca Ski (middle) and SiHa (bottom) cells treated or not with 0.25 μM of 5azadC for 96h. **(B)** Ca Ski and SiHa cells were mock treated or treated with 5azadC. Protein extracts were analyzed by immunoblotting for the detection of E6Fl protein. Pos Ctrl: positive control (top). The data are presented as mean values from at least three independent experiments. Error bars represent the standard deviation and *p* values were calculated by performing Mann-Whitney test: * p < 0.05 (bottom). **(C)** Expression of E6Fl mRNA was quantified by RT-qPCR and normalized to the β2M RNA level in Ca Ski and SiHa cells treated or not with 0.25 μM of 5azadC for 96h. The data are presented as mean values from at least three independent experiments. Error bars represent the standard deviation and *p* values were calculated by performing Mann-Whitney test: *** p < 0.001. **(D)** Representative relative luciferase units (RLU = reporter firefly/renilla control ratios) assessing the presence of E2 protein in Ca Ski and SiHa cells transfected with pGL3TKminLuc or pGL3E2BSTKminLuc for 24h. The transfection of pciNeoE2 served as a positive control.

Then, the methylation status of proximal E2BS located in the LCR was investigated in both cell lines treated with 5azadC using HRM PCR as we previously described [17]. Untreated Ca Ski and SiHa cells were assigned to the 75% and 0% methylation profiles respectively. The proportion of methylated proximal E2BS decreased to 25% in Ca Ski cells exposed to 5azadC for 96h. As expected, this proportion was not modified in SiHa cells that were assigned to the 0% methylation profile (Figure 1A). These data showed that 5azadC efficiently reduced the methylation level of proximal E2BS in Ca Ski cells.

Next, E6 protein and full length E6-encoding mRNA (E6Fl) levels were analyzed using Western blotting and RT-qPCR in Ca Ski and SiHa cells treated with 5azadC at 0.25 μM for 96h. The decrease in E6 protein in treated cells (Figure 1B) was confirmed and a decrease in E6Fl mRNA levels was observed in both cell lines compared to control cells (Figure 1C).

Taking into account that E2 is the major regulator of the p97 promoter, the loss of E6 protein expression could be the consequence of the E2 interaction with demethylated E2BS at least in Ca Ski cells that harbor intact E2 ORFs. Regarding SiHa cells, E2 independent mechanism(s) likely decrease E6 expression in cells harboring demethylated DNA due to the disruption of the E2 gene. To address whether Ca Ski cells expressed constitutively the E2 protein, a luciferase reporter construct under the control of E2BS (pGL3E2BSTKminLuc) was transfected in these cells. SiHa cells were used as negative control. The co-transfection of pciNeoE2 was used as a positive control. As shown in Figure 1D, no luminescence was emitted by cells transfected with pGL3TKminLuc or pGL3E2BSTKminLuc. Luciferase activity was observed only in Ca Ski and SiHa cells co-transfected with pGL3E2BSTKmin Luc and pciNeoE2. These data indicate that the E2 protein is either not expressed or expressed as a non-functional protein in Ca Ski and SiHa cell lines, suggesting that the repression of E6 expression by 5azadC was not due to E2 in Ca Ski cells. Thus, we explored other possible processes for the effect of 5azadC treatment on E6 repression.

### 5azadC treatment does not modify the expression of cellular transcription factors known to activate or inhibit the p97 promoter

**Figure 2:**
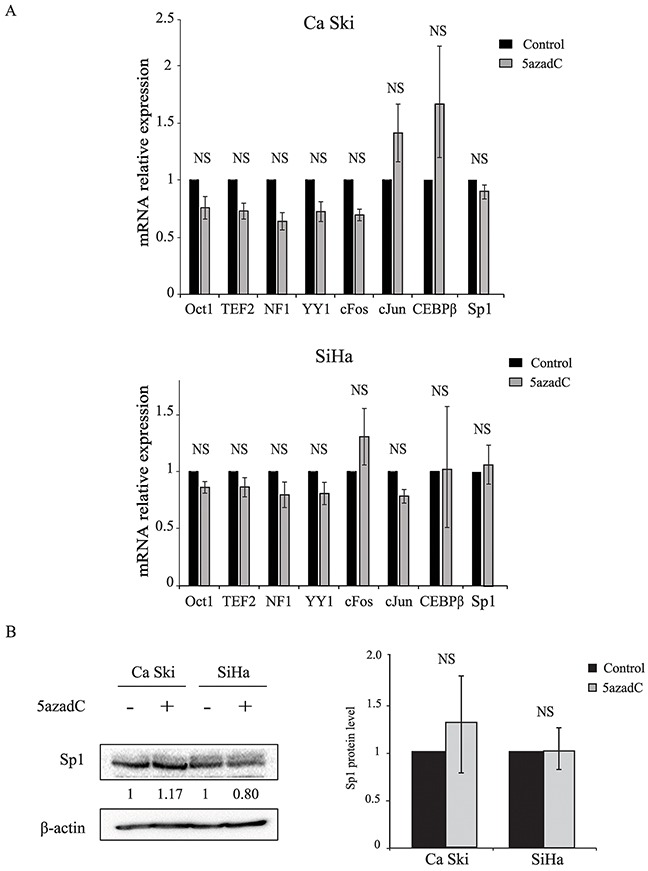
Effect of 5azadC treatment on cellular transcription factor expression **(A)** Total RNAs were extracted from Ca Ski and SiHa cells treated or not with 5azadC for 96h and the relative expression of Oct1, TEF-2, NF1, YY1, cFos, cJun, CEBPβ and Sp1 mRNA was measured by RT-qPCR and normalized to β2M mRNA level. The data are presented as mean values from at least three independent experiments. Error bars represent the standard deviation and *p* values were calculated by performing Mann-Whitney test: NS: no significant. **(B)** Mock treated and 5azadC-treated (0.25 μM for 96h) Ca Ski and SiHa cells were lysed and the protein fraction was assayed for the presence of Sp1 by Western blotting. β-actin served as a loading control. A representative blot is shown; the values of the densitometric analysis were normalized with β-actin (left). The data are presented as mean values from at least three independent experiments. Error bars represent the standard deviation and *p* values were calculated by performing Mann-Whitney test: NS: no significant (right).

Some ubiquitous cellular transcription factors are known to activate or inhibit HPV16 p97 promoter. RT-qPCR analysis of Oct1, TEF-2, NF1, YY1, cFos, cJun, CEBPβ and Sp1 mRNA expression showed no significant difference between control and 5azadC-treated cells (Figure 2A). Given that Sp1 plays a critical role in p97 activation, the amount of Sp1 protein was determined consecutive to 5azadC treatment, and no significant difference was observed between treated and control cells (1.3- and 1.0-fold increase in Ca Ski and SiHa cells respectively) (Figure 2B). These results suggest that 5azadC does not modify the expression of the main transcription factors involved in p97 regulation.

### E6 RNA splicing pattern is not modified but E6 mRNA stability is decreased after 5azadC treatment

**Figure 3:**
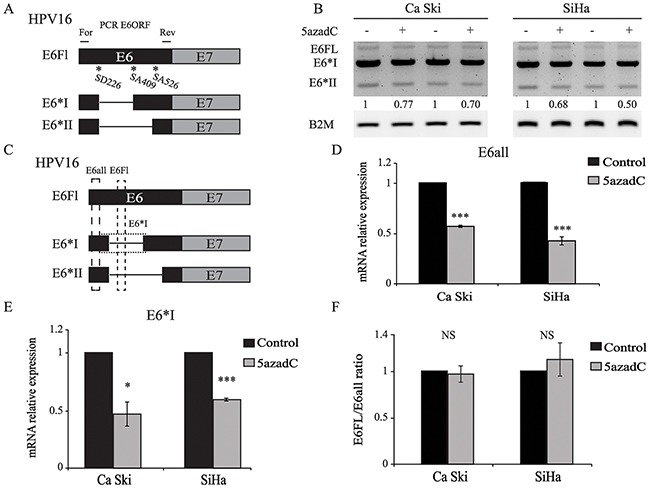
E6 RNA splicing pattern is not modified by 5azadC treatment **(A)** Splicing pattern of E6 RNAs. The position of forward (For) and reverse (Rev) primers is shown. **(B)** Total RNAs were extracted from mock treated and 5azadC-treated (0.25 μM for 96h) Ca Ski and SiHa cells. Two representative and independent RT-PCR analysis of the E6ORF show E6Fl, E6*I and E6*II mRNA after gel electrophoresis. Semi-quantitation of all E6 transcripts was carried out by densitometry analysis normalized to β2M mRNA level. **(C)** Schematic view of E6all, E6Fl and E6*I RT-qPCR products. **(D, E)** E6all **(D)** and E6*I **(E)** mRNA were quantified by RT-qPCR and normalized to β2M mRNA level in three independent experiments. **(F)** E6Fl/E6all mRNA ratios were calculated to reveal changes in the splicing pattern of E6 transcripts. The data are presented as mean values from at least three independent experiments. Error bars represent the standard deviation and *p* values were calculated by performing Mann-Whitney test: * p < 0.05; *** p < 0.001; NS: not significant.

HPV16 gene expression is also post-transcriptionally regulated by RNA splicing. Recently, next generation sequencing allowed the identification of at least 20 polycistronic HPV transcripts due to alternative splicing and expressed in high grade cervical lesions [29]. At least three E6 RNAs, commonly named E6Fl, E6*I and E6*II can be generated by alternative splicing (Figure 3A). In order to assess whether 5azadC modifies the splicing pattern of E6 transcripts, unspliced and spliced E6 RNAs were analyzed by RT-PCR using primers specifically designed to amplify simultaneously E6Fl, E6*I and E6*II transcripts. These transcripts were expressed in Ca Ski and SiHa cells and 5azadC treatment did not modify the splicing profile of E6 transcripts (Figure 3B). RT-qPCR was used to independently quantify either all E6 transcripts (E6all), or E6Fl and E6*I individually (Figure 3C). As expected, the level of E6all RNA was lower in treated cells compared to control cells (Figure 3D). The same trend was observed for E6*I RNA level (Figure 3E). Finally, the E6Fl/E6all mRNA ratios calculated in control and treated cells were very similar, ruling out the hypothesis that 5azadC treatment modified the splicing pattern of E6 transcripts (Figure 3F).

**Figure 4:**
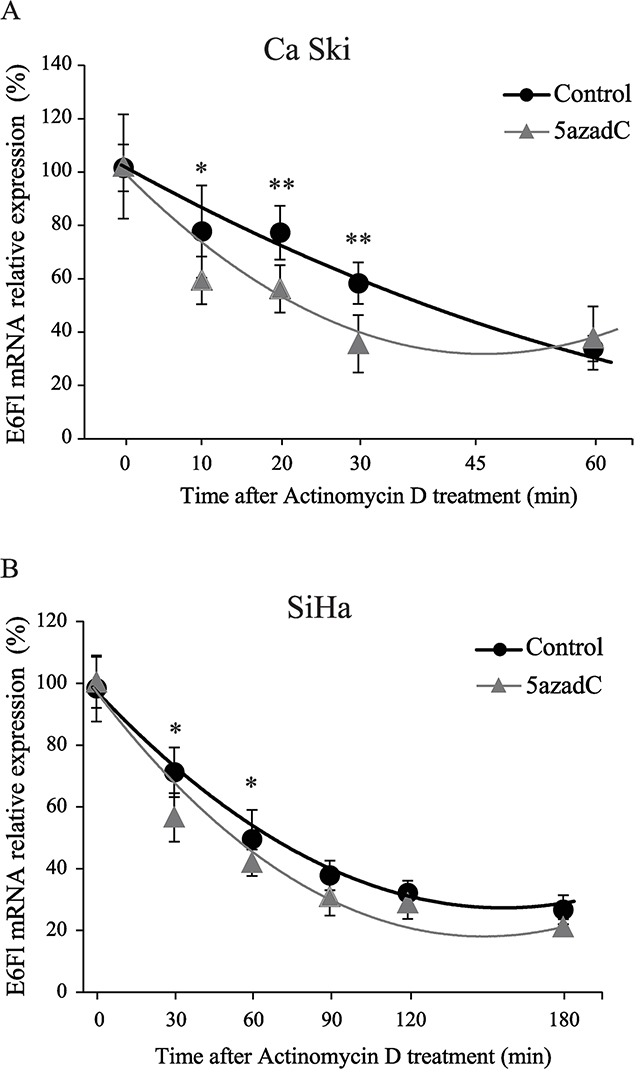
Destabilization of E6 transcripts after 5azadC treatment **(A, B)** RNAs were extracted from Ca Ski **(A)** and SiHa **(B)** cells treated or not with 5azadC at 0.25 μM during 96h and treated with 4 μg/mL actinomycin D. Relative expression of E6Fl mRNA was determined by RT-qPCR and normalized to β2M mRNA level. The diagram shows the mean values for at least three independent experiments and the standard deviation. Mann-Whitney`s test was performed to calculate *p* values using DMSO treated cells as a reference control. *p < 0.05, **p < 0.01.

To assess whether 5azadC affects the stability of E6Fl RNA, we monitored RNA level during a time-course treatment with actinomycin D. As shown in Figure 4A, the half-life of E6Fl mRNA was significantly decreased from 40 min to 20 min in Ca Ski cells after 5azadC treatment. In SiHa cells, a slight but significant decrease of E6Fl mRNA half-life was observed from 65 min to 51 min (Figure 4B). Taken together, these results suggest that the modulation of E6 expression might be partly due to a destabilization of E6 transcripts.

### Demethylation induces an increased expression of miR-375 which correlates with E6 RNA destabilization and protein loss

**Figure 5:**
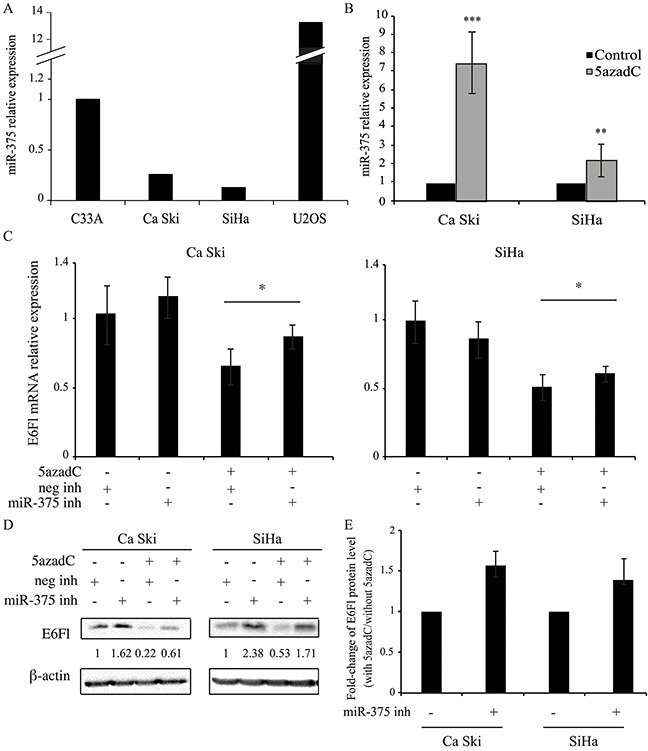
miR-375 is involved in the decrease of E6 expression **(A, B)** miR-375 relative expression was quantified by RT-qPCR and normalized to U6 snRNA level in C33A, Ca Ski, SiHa and U2OS cells **(A)** and in Ca Ski and SiHa cells **(B)** treated or not with 0.25 μM of 5azadC for 96h. The data are presented as mean values from at least three independent experiments. Error bars represent the standard deviation and *p* values were calculated by performing Mann-Whitney test: ** p < 0.01; *** p < 0.001 (B) **(C, D)** Ca Ski and SiHa cells were treated with 5azadC at 0.25 μM during 96h. Cells were transfected with miRIDIAN microRNA negative control or miR-375 inhibitors at 200 nM during the last 24h. E6Fl mRNAs were analyzed by RT-qPCR and normalized to β2M mRNA level **(C)** and E6 proteins were analyzed by Western blotting and normalized to β-actin protein level **(D)**. The data are presented as mean values from at least three independent experiments. Error bars represent the standard deviation and *p* values were calculated by performing Mann-Whitney test between 5azadC-treated cells +/− miR-375 inhibitor: * p < 0.05 **(E)** Fold-change in E6Fl protein levels induced by miR-375 inhibitor was calculated in 5azadC-treated cells compared to control cells. The data are presented as mean values from at least three independent experiments. Error bars represent the standard deviation and *p* values were calculated by performing Mann-Whitney test: *p*=0.06 for both cells.

Recently, it was reported that miR-375 mimics induced the suppression of E6 expression in HPV positive cell lines [24, 30]. First the miR-375 level was measured in HPV16 positive cell lines (CaSki and SiHa) and in HPV negative cell lines (C33A and U2OS). As shown in Figure 5A, miR-375 expression was low in Ca Ski and SiHa cells compared to the HPV negative cervical cancer cell line C33A or to the osteosarcoma cell line U2OS.

To determine whether miR-375 level was modulated after DNA demethylation, RT-qPCR was performed in HPV16 positive cells treated or not with 5azadC. Interestingly, after 5azadC exposure, the miR-375 expression was upregulated 7.4-fold and 2.2-fold in CaSki and SiHa cells respectively (Figure 5B).

To confirm the implication of miR-375 in HPV16 transcript repression, the effect of a miR-375 inhibitor was tested in HPV16 positive cells. In the absence of 5azadC, no significant change was observed in E6Fl mRNA level after addition of miR-375 inhibitor (Figure 5C) while the corresponding protein was increased (Figure 5D). As expected, decreased expression of E6Fl at both mRNA (Figure 5C) and protein (Figure 5D) levels was observed in 5azadC-treated cells. Interestingly, we observed that the miR-375 inhibitor restored E6Fl mRNA expression in 5azadC-treated cells, from 0.65 to 0.86 in Ca Ski cells and from 0.51 to 0.61 in SiHa cells (Figure 5C). This increase was also observed at the protein level (Figure 5D). Then, the E6Fl protein rescue was estimated in cells transfected with the miR-375 inhibitor in the presence or not of 5azadC. As shown in Figure 5E, miR-375 inhibitor transfection induced an increase of 1.52- and 1.35-fold in E6 expression in Ca Ski and SiHa cells respectively compared to control cells at the limit of significance (*p*=0.06).

Altogether, these data show that global DNA demethylation by 5azadC upregulates miR-375 expression, which consequently likely leads to destabilization of HPV16 transcripts and loss of E6 protein expression.

## DISCUSSION

In this study, we showed that the treatment of HPV16 positive Ca Ski and SiHa cells with the demethylating agent 5azadC induces repression of E6 expression at both mRNA and protein levels. This result was somewhat unexpected for Ca Ski cells. Indeed, this cell line presents nearly 500 integrated HPV16 genomes in head-to-tail concatemers and a highly methylated LCR (>80%), especially on the 5 CpG involved in the regulation of early viral gene expression and located in the two proximal E2BS [17]. Usually, high levels of CpG methylation contribute to gene silencing by preventing the binding of transcription factors to specific regulatory sequences or by inducing chromatin compaction [31]. In this regard, it has been demonstrated by *in vitro* studies that LCR methylation can silence p97 promoter and limit the expression of early genes [32, 33]. Thus, it would have been consistent to observe increased expression of E6 after 5azadC treatment. Indeed, proximal E2BS were effectively demethylated in treated Ca Ski cells making promoter sequences accessible to binding by transcription factors. Because E2 is the main repressor of p97, we sought to determine whether this protein was expressed in Ca Ski cells. The use of a very sensitive luciferase test demonstrated the absence of functional E2 protein as previously described [34]. Furthermore, E6 was also downregulated in SiHa cells which exhibit disrupted E2 ORFs and unmethylated E2BS. Thus, the repression of E6 in response to DNA demethylation by 5azadC treatment appears independent of E2 protein. Hence, we explored other possible mechanisms for the effect of 5azadC treatment on E6 repression.

We focused our attention on the cellular transcription factors (Oct1, TEF-2, NF1, YY1, cFos, cJun, CEBPβ and Sp1) known to bind sequences that regulate HPV16 early gene expression [6, 35]. Few data concerning epigenetic regulation of transcription factors are available. Some studies have reported that 5azadC induces a decrease in AP-1 expression in bone marrow mononuclear cells [36], an increase in CEBPα but a decrease in CEBPγ expression in acute myeloid leukemia cells [37] and an increase in CEBPα expression in three HPV-negative head and neck squamous cell carcinoma cell lines [38]. In Ca Ski and SiHa cells, none of the studied transcription factors (either activator or repressor) appeared significantly increased or decreased by 5azadC treatment. Post-translational modifications of transcription factors are known to modulate their activity and/or stability [39]. Such modifications were not investigated in the present study and it cannot be excluded that they can be altered by DNA demethylation leading to variations in the function of these central regulators of gene expression.

HPV16 transcripts are polycistronic mRNAs submitted to alternative splicing. Interestingly, Piotrawska et al. showed that 5azadC contributes to the variable expression of splice variants of Glucocorticoid Receptor alpha and beta, which was accompanied by increased expression of the serine/arginine splicing factor 2 (SRSF2) in breast carcinoma cells [40]. In our cellular models, the different splice variants of E6 were decreased after 5azadC treatment and no change in the E6FL/E6all mRNA ratio was observed, indicating that demethylation of DNA did not favor the splicing of E6Fl mRNA into small spliced isoforms E6*I or E6*II. McFarlane *et al*. showed that the depletion of the splicing factor SRSF2 in Ca Ski cells favored the reduction of E6/E7 transcripts [41]. They demonstrated that it was not due to down-modulation of transcription initiation or elongation, but rather to mRNA destabilization, and they proposed that SRSF2 might protect viral mRNA from non-sense mediated RNA decay (NMD).

The exposure of Ca Ski and SiHa cells to actinomycin D made it possible to demonstrate that the stability of E6Fl mRNA was reduced after 5azadC treatment. Similarly, Luczak et al. used the HDAC inhibitor apicidin, another epidrug, and observed a decrease of E6/E7 transcript stability in SiHa cells. However, the molecular mechanism underlying transcript destabilization by apicidin was not investigated in Luczak's study [42]. Recently, miR-375 has been shown to negatively regulate E6/E7 transcripts in HPV16 positive carcinoma cell lines [24, 30]. Consistent with these observations, a 2.2 to 7.4-fold increase in miR-375 expression was observed in 5azadC-treated SiHa and Ca Ski cells, respectively, suggesting that miR-375 could play a role in E6 mRNA destabilization. In treated Ca Ski cells, the E6Fl mRNA half-life decreased by half (from 40 to 20 min) compared to control cells. There was only a 22% decrease (65 to 51 min) in SiHa cells, suggesting that E6Fl mRNA destabilization was less efficient in this cell line as compared to Ca Ski cells. This may be due to the lower level of miR-375 measured in SiHa cells compared to Ca Ski cells after 5azadC treatment. Interestingly, Wilting et al. and Stich et al. have demonstrated that the treatment of HPV16 positive cervical and head and neck cancer cell lines by 5azadC led to miR-375 promoter demethylation, an observation consistent with a direct effect of 5azadC in miR-375 overexpression [27, 30]. In this report, we confirmed that the treatment with a demethylating agent increases the expression of miR-375 in SiHa cells and to a greater extent in Ca Ski cells. Moreover, the use of miR-375 inhibitor supported a role for miR-375 in the repression of E6 by 5azadC since it reverses the effect of the demethylating agent on E6 expression. These data are consistent with those obtained by Jung *et al*. and Stich et al. who used miR-375 mimic to repress the expression of E6/E7 RNA and E7 protein in HPV16 positive carcinoma cell lines [24, 30].

The weaker effect of miR-375 inhibitor on E6Fl mRNA compared to the effect on protein expression could be related to the mechanisms by which miRNAs regulate gene expression. Indeed, miRNAs can induce translational repression and/or mRNA decay, and miRNA-mediated silencing depends on the concentration of RNA-induced silencing complex (RISC) components, the sequence of target mRNAs (number of miRNA-binding sites) and cell types. Moreover, recent genome-wide analyses support the model that miRNAs induce translational repression before mRNA decay [43]. Thus, miR-375 inhibitor probably first modulate translation repression induced by miR-375, while an increase in E6Fl mRNA expression would require much longer exposure to miR-375 inhibitor. Additional studies on RISC composition and function following 5azadC treatment could help to better understand the role of miR-375 in E6 repression.

The miR-375 exhibits tumor suppressor activity through inhibition of proliferation, invasion and motility [44]. E6 and E7 are known to modulate the activity and the expression of DNMT1 and histone methyltransferase to down-regulate the expression of numerous tumor suppressor genes [45–48] or miRNA through promoter methylation or cell signaling pathway [49–51]. Thus, miR-375 promoter methylation may be one strategy developed by HPV16 to allow efficient and sustained expression of E6/E7 oncogenes to functionally inactivate anti-proliferative and anti-apoptotic signaling pathways. Furthermore, Liu et al. recently showed that E6 participates in the silencing of miR-375 expression through overexpression of DNMT1, which in turn methylates miR-375 promoter [52].

Nonetheless, our study presents some limitations. Experiments addressed the role of miR-375 only, but other miRNAs (miR-122, miR-875 and miR-3144) have been shown to repress the expression of E6/E7 transcripts [53, 54]. Therefore, it cannot be excluded that one of these miRNAs participates in E6 repression after 5azadC treatment. This would explain why E6 expression is not totally rescued following miR-375 inhibitor treatment. The conclusions presented here rely on the analysis of two cervical cancer cell lines. We obtained similar results with a HPV16-positive head and neck cancer cell line regarding the down-regulation of E6 expression at RNA and protein levels following 5azadC treatment (data not shown). Our results are consistent with those recently published by Stich et al. who also reported a 2- to 3-fold reduction in miR-375 promoter methylation following the treatment of HPV16-positive cell lines with 5azadC [30], an observation consistent with a direct effect of this demethylating agent on miR-375 up-regulation. The effect of 5azadC treatment on cell viability was not investigated. Since it was previously shown that E6 was repressed in apoptotic HPV16-positive cells following staurosporine- or isoliquiritigenin exposure [55, 56], we can hypothesize that 5azadC could favor cell death of HPV16-positive cells.

To conclude, in this study we have shown that HPV16 E6 expression was down-regulated in Ca Ski and SiHa cells at mRNA and protein levels after 5azadC treatment. This repression was not associated with modifications of expression of ubiquitous cellular transcription factors known to bind the LCR. Moreover, the splicing pattern of E6 mRNA was not altered. Rather, we evidenced a destabilization of E6 mRNA thanks to miR-375 upregulation. Further investigations are necessary to better understand the relationship between E6 and miR-375 regulation, to gain further insight into HPV16-associated carcinogenesis. Moreover, the development of targeted therapies based on miRNA mimics [57] or epidrugs [58, 59] will pave the way for the emergence of new therapeutic strategies for HPV-associated cancers.

## MATERIALS AND METHODS

### Cell culture

Ca Ski cells (ATCC, Manassas, VA, USA) were maintained in RPMI-1640 medium while SiHa, C33-A and U2OS cells (ATCC) were maintained in Dulbecco's modified Eagle medium (DMEM), both supplemented with 10% (v/v) fetal bovine serum (Lonza, Basel, Switzerland) in a humidified CO_2_ incubator at 37°C. Ca Ski and SiHa cells, two HPV16 cell lines, were treated with 5-aza-2′-deoxycytidine (5azadC) (Epigentek, Farmingdale, NY, USA) at a final concentration of 0.25 μM for 48h or 96h. The treatment medium was renewed every day.

### DNA extraction and bisulfite conversion

DNA was extracted using QiAmp DNA Mini kit (QIAGEN, Courtaboeuf, France) according to the manufacturer's instructions and converted using Cells to CpG bisulfite conversion kit (Life Technologies). Briefly, 500 ng of DNA were denatured at 50°C for 10 min with the denaturation agent. One hundred microliters of reconstituted conversion reagent were added to the denatured DNA and the solution was incubated for two cycles of 30 min at 65°C and 1.5 min at 95°C followed by 30 min at 65°C. The converted DNA was purified to remove salts and sulfonic groups and eluted in 40 μL of elution buffer.

### HRM PCR

The HRM PCR assesses the 5 CpG islands methylation located on the proximal E2BS of HPV16 p97 promoter. The experiment was performed using the ABI 7500 Fast real-time PCR system and Melt Doctor HRM Master mix (Life Technologies) as we previously described in Jacquin *et al*., 2013 [18].

### Plasmid transfection and luciferase assay

Ca Ski and SiHa cells were transfected with the mixture of each vector (pciNeo+/−E2 and pGL3TKmin Luc+/−E2BS) using JetPEI® transfection reagent (Polyplus transfection, Illkirch, France) according to the manufacturer's recommendations. Cells were lysed by Lysis reagent 1X of Dual-Luciferase Reporter Assay System kit (Promega, Charbinnières les bains, France). Lysates were mixed with the Luciferase assay reagent and the luminescence was recorded. Luminescence signals were then stopped through Stop and Glo reagent and Renilla signals were measured to normalize luciferase luminescence.

### RNA extraction and reverse transcription

Total RNAs were extracted by Trizol®-chloroform method (Sigma-Aldrich, Saint Quentin Fallavier, France). cDNA were synthetized using the Maxima First Strand cDNA Synthesis kit (Thermo Scientific, Illkirch, France) according to the manufacturer's recommendations.

### qPCR

Viral (E6 Full length, E6*I, E6all), cellular transcription factors (TF) (Oct1, cFos, cJun, TEF-2, NF1, CEBPβ, YY1 and Sp1) cDNA were quantified by real-time PCR using the ABI 7500 Real-Time PCR System (Applied Biosystems, Saint Aubin, France) with the SYBR Green real time PCR master mix (Life Technologies, Saint Aubin, France). The specific primer sequences of selected genes are shown in Table 1. Thermal cycling for all qPCR consisted in 95°C 5 min followed by 40 cycles of 30 s at 95°C and 1 min at 60°C. mRNA levels were normalized to β2 microglobulin using the 2^−ΔΔCt^ method.

**Table 1:** Primer sequences

Gene	Forward (5′->3′)	Reverse (5′->3′)	Amplicon
**E6Fl**	CACAGGAGCGACCCAGAAA	CCCGAAAAGCAAAGTCATATACC	131 bp
E6*I	CTGCGACGTGAGGTGTATTA	TGTCCAGGTGTCTTTGCTTT	68 bp
**E6all**	GAGAACTGCAATGTTTCAGGACC	TGTATAGTTGTTTGCAGCTCTGTGC	80 bp
**B2M**	GATGAGTATGCCGTGTG	CAATCCAAATGCGGCATCT	114 bp
**Oct1**	CAGATCGCACAGGATCTTCA	GGATGCACCAACACAAACTG	80 bp
**TEF-2**	AAAGCTCCCACTTGAAAGCA	AACTTCCATGTGCACCCTTC	85 bp
**NF1**	GTGTTCCATGTGCGATTACG	TCATGTGCCTTTTCAGCTTG	140 bp
**YY1**	ACCTGGCATTGACCTCTCAG	TTCTCATGGCCGAGTTATCC	149 bp
**cFos**	AGAATCCGAAGGGAAAGGAA	CTTCTCCTTCAGCAGGTTGG	150 bp
**cJun**	TGACTGCAAAGATGGAAACG	CAGGGTCATGCTCTGTTTCA	119 bp
**CEBPβ**	TACTACGAGGCGGACTGCTT	AGGTACGGGCTGAAGTCGAT	149 bp
**U6**	CGCTTCGGCAGCACATATAC	AAAATATGGAACGCTTCACGA	100 bp

### E6ORF PCR

E6ORF_For5′-ATGCACCAAAAGAGAACTGC-3′ and E6ORF_Rev5′-TTACAGCTGGGTTTCTCTACGTGT-3′ primers were designed to target the ORF of E6Fl, E6*I and E6*II. PCR were performed with 0.5 μM of each primer, 0.625 U DreamTaq Polymerase (ThermoFisher Scientific, Waltham, USA), DreamTaq Buffer 1X and 2.5 μL of cDNA. Amplification consisted in a first step at 95°C for 2 min followed by 30 cycles of denaturation at 95°C (30 sec), hybridization at 50°C (20 sec), elongation at 72°C (30 sec) and a final step at 72°C for 5 min. After electrophoresis on a 2% agarose gel, PCR product levels were normalized to β2 microglobulin using densitometry.

### Quantification of miR-375

After RNA extraction, reverse transcription was carried out using the TaqMan MicroRNA Reverse Transcription kit (Life Technologies). Relative quantities of miR-375 (Cat#4427975, Life Technologies) were determined with the 7500 Real-Time PCR System by using TaqMan® microRNA Assays and TaqMan Universal Master Mix II (Life Technologies). miR-375 expression was normalized by the endogenous control U6 snRNA (Primers: Table 1) using the 2^−ΔΔCt^ method.

### Suppression of miR-375 activity

Ca Ski and SiHa cells were treated with 5azadC at 0.25 μM for 96h. During the last 24h, cells were transfected with miRIDIAN microRNA Human miR-375 hairpin inhibitor (IH-3006882-07) or miRIDIAN miRNA Hairpin Inhibitor Negative Control (IN-001005-01) (Dharmacon, GE Healthcare, LaFayette, USA) at 200 nM with Lipofectamine 2000 (Invitrogen, Villebon sur Yvette, France) according to the manufacturer's recommendations. Cells were then processed for analysis of E6Fl mRNA and protein expression.

### Western blotting

Proteins were extracted with radioimmuno-precipitation assay lysis buffer (50 mM Tris/HCl, pH 7.4, 150 mM NaCl, 0.1% SDS, 1 % Nonidet P-40, 0.5 % Na deoxycholate, 1 mM EDTA), protease inhibitor (30 μg/mL) (Roche Diagnostics, Meylan, France). After sonication, protein concentrations were determined using the BioRad Protein assay (Biorad, Marnes-la-Coquette, France) according to the manufacturer's recommendations. Each sample was subjected to SDS-PAGE and then transferred onto Hybond TM-P polyvinylidene difluoride membranes (GE Healthcare, Amersham, England) which were blocked with 5% nonfat milk overnight at 4°C under constant shaking. Membranes were then incubated with primary antibodies (see below), washed and incubated with anti-mouse or anti-rabbit immunoglobulin antibodies conjugated with horseradish-peroxydase. The following primary antibodies were used for this study: 2E3F8 anti-E6HPV16 (Euromedex, Souffelweyersheim, France); DO7 anti-p53 (BD Biosciences, Le Pont de Claix, France); ABE135 anti-Sp1 (Millipore, Saint-Quentin-en-Yvelines, France) and AC15 anti-βactin (Sigma-Aldrich) antibodies. The immune complexes were revealed by chemiluminescence with Pierce ECL2 Western blotting Substrate (Thermo Scientific) using ChemiDoc XRS+ with image lab software (Biorad).

### Statistical analyses

Data are presented as mean ± standard deviation (SD). Group comparisons were performed using the Mann-Whitney test. A *p* value <0.05 was considered as statistically significant; *, **, and *** illustrate significance at 0.05, 0.01, and 0.001 levels respectively. A one-way ANOVA has been used to correct for multiple testing.

## SUPPLEMENTARY FIGURE


