# Supplementary material for: 5azadC treatment upregulates miR-375 level and represses HPV16 E6 expression
Source: Oncotarget. 2017 May 2;8(28):46163–76. doi: 10.18632/oncotarget.17575 (PMC5542257; doi:10.18632/oncotarget.17575)
Supplement: Supplementary file 1 [file oncotarget-08-46163-s001.pdf]

5azadC treatment upregulates miR-375 level and represses HPV16 E6 expression

SUPPLEMENTARY FIGURE

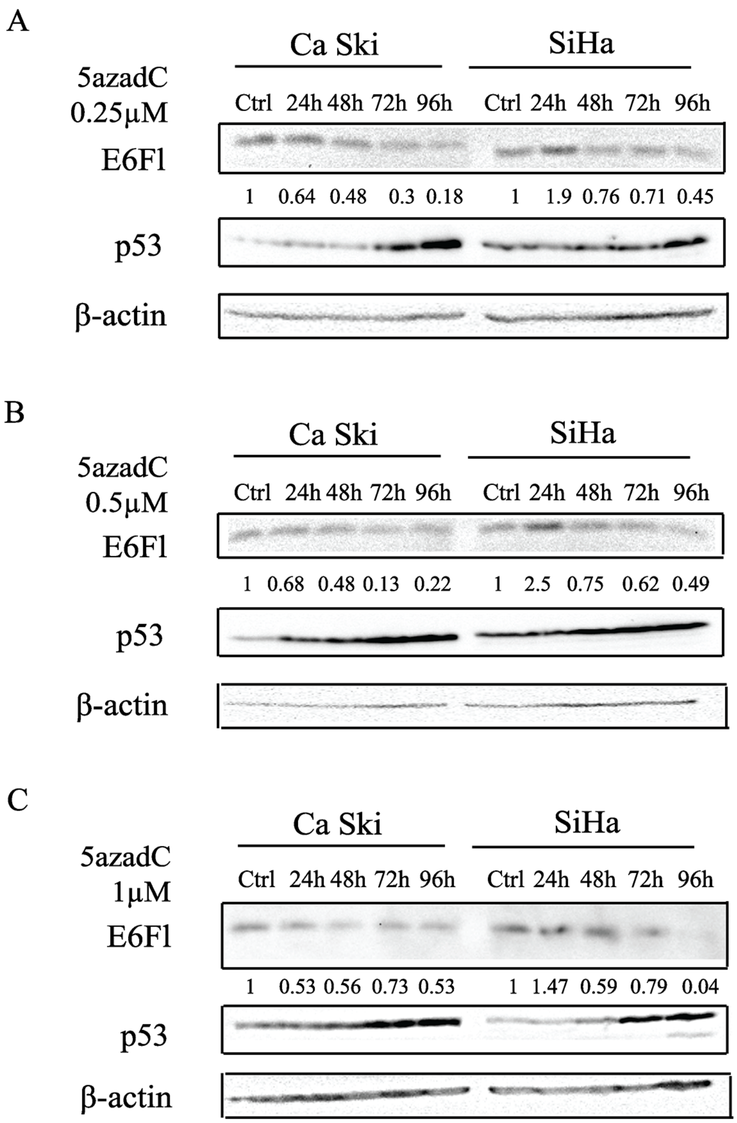

Supplementary Figure 1: Ca Ski and SiHa cells were treated with 0.25 µM **(A)**, 0.5 µM **(B)** and 1 µM **(C)** of 5azadC for 24 to 96h. Cells were lysed and protein extracts were assayed by Western blotting to assess the levels of E6FI and p53 proteins. β-actin served as a loading control.
